# A new and promiscuous α/β hydrolase from *Acinetobacter tandoii* DSM 14970^ T^ inactivates the mycotoxin ochratoxin A

**DOI:** 10.1007/s00253-024-13073-x

**Published:** 2024-02-23

**Authors:** Ana Sánchez-Arroyo, Laura Plaza-Vinuesa, María Claudia Abeijón-Mukdsi, Blanca de las Rivas, José Miguel Mancheño, Rosario Muñoz

**Affiliations:** 1 Bacterial Biotechnology, Institute of Food Science, Technology and Nutrition (ICTAN), CSIC, José Antonio Novais 6, 28040 Madrid, Spain; 2 Department of Crystallography and Structural Biology, Institute of Physical Chemistry Blas Cabrera, CSIC, Serrano 119, 28006 Madrid, Spain

**Keywords:** Amidohydrolase, Esterase, Mycotoxin, Ochratoxin A, Promiscuous enzymes

## Abstract

**Abstract:**

The presence of ochratoxin A (OTA) in food and feed represents a serious concern since it raises severe health implications. Bacterial strains of the *Acinetobacter* genus hydrolyse the amide bond of OTA yielding non-toxic OTα and L-β-phenylalanine; in particular, the carboxypeptidase PJ15_1540 from *Acinetobacter* sp. *neg1* has been identified as an OTA-degrading enzyme. Here, we describe the ability to transform OTA of cell-free protein extracts from *Acinetobacter tandoii* DSM 14970^ T^, a strain isolated from sludge plants, and also report on the finding of a new and promiscuous α/β hydrolase (ABH), with close homologs highly distributed within the *Acinetobacter* genus. ABH from *A. tandoii* (*At*ABH) exhibited amidase activity against OTA and OTB mycotoxins, as well as against several carboxypeptidase substrates. The predicted structure of *At*ABH reveals an α/β hydrolase core composed of a parallel, six-stranded β-sheet, with a large cap domain similar to the marine esterase EprEst. Further biochemical analyses of *At*ABH reveal that it is an efficient esterase with a similar specificity profile as EprEst. Molecular docking studies rendered a consistent OTA-binding mode. We proposed a potential procedure for preparing new OTA-degrading enzymes starting from promiscuous α/β hydrolases based on our results.

**Key points:**

*• AtABH is a promiscuous αβ hydrolase with both esterase and amidohydrolase activities*

*• AtABH hydrolyses the amide bond of ochratoxin A rendering nontoxic OTα*

*• Promiscuous αβ hydrolases are a possible source of new OTA-degrading enzymes*

**Supplementary Information:**

The online version contains supplementary material available at 10.1007/s00253-024-13073-x.

## Introduction

Ochratoxin A (OTA) is one of the most prevalent mycotoxins that contaminate food and feed products around the world (Liuzzi et al. 2017; Marin et al. 2013). OTA can have a variety of noxious effects both in humans and in other animals (Gan et al. 2017), although it is best known for its nephrotoxic effects (Pfohl-Leszkowicz and Manderville 2007). While a positive association between some mycotoxins, such as aflatoxin, and cancer has been verified (Claeys et al. 2020), the potential link between OTA exposure and cancer risk is not currently established and needs further epidemiological studies. Nonetheless, to avoid its toxic effects, the levels of OTA must be reduced as much as possible. Up to now, the most important measures to control OTA levels have focused on avoiding fungal growth and OTA production (Amézqueta et al. 2009). In this regard, biological approaches have increasingly been considered as an alternative to physical and chemical treatments (Abraham et al. 2022), and currently, it has been reported that microorganisms that adsorb and/or degrade OTA and even some practical procedures have been developed thereafter (Ndiaye et al. 2022). However, some reports describing microbial OTA-degrading activity do not discriminate between physical adsorption or enzymatic degradation. In this line, it is now generally accepted that whereas detoxification by lactic acid bacteria mainly proceeds through adsorption of OTA to their cell walls (Del Prete et al. 2007), other organisms, including actinobacteria, bacteria, filamentous fungi, and yeast (Xu et al. 2022), transform OTA through hydrolysis of its amide bond to generate OTα and L-β-phenylalanine, virtually non-toxic compounds.

Only a handful of enzymes involved in the degradation of OTA have been isolated and characterized in detail. The first reported microbial (fungal) ochratoxinase was an *Aspergillus niger* amidase (Dobritzsch et al. 2014). Later, bacterial proteins annotated as putative carboxypeptidases from *Bacillus amyloliquefaciens* ASAG1 (Chang et al. 2015), *Bacillus subtilis* CW14 (Hu et al. 2018; Xu et al. 2021), *Lysobacter* sp. CW239 (Wei et al. 2020), and *Acinetobacter* sp. *neg1* (Liuzzi et al. 2017) were described. Moreover, bacterial enzymes able to efficiently transform OTA have been identified in *Alcaligenes faecalis* DSM 16503 (Zhang et al. 2019), and *Stenotrophomonas* sp. CW117 (Chen et al. 2022; Luo et al. 2022). In this regard, we have recently reported that the salicylate 1,2-dioxygenase from *Pseudaminobacter salicylatoxidans* inactivates OTA acting as an amidohydrolase and proposed that metallocarboxypeptidases may be a rich source of potential OTA-degrading enzymes (Sánchez-Arroyo et al. 2023). In that study, with the aid of currently available, powerful molecular docking approaches, we proposed an OTA-binding mode and a catalytic mechanism of OTA hydrolysis, which is consistent with those from metalocarboxypeptidases. This very same in silico approach is being used here, combined with the use of AlphaFold2 (Jumper et al. 2021) for predicting the structure of *At*ABH. In this sense, the accuracy of the predicted models produced by this astonishingly powerful program (Pereira et al. 2021) is such that it has changed the scene of structural biology, and they are used for the generation of convincing hypotheses about interacting proteins (Tunyasuvunakool et al. 2021) or structure solution by molecular replacement (McCoy et al. 2022; see Read et al. 2023).

Bacteria from the genus *Acinetobacter* are widely distributed in nature, being present in soils, freshwater, oceans, sediments, and contaminated sites (Jung and Park 2015). *Acinetobacter* species are metabolically versatile in that they possess numerous catabolic routes aimed at the degradation of compounds from plants, such as routes for the degradation of various long-chain dicarboxylic acids and aromatic and hydroxylated aromatic compounds. For these reasons, the genus *Acinetobacter* has attracted significant attention both in the scientific and the biotechnological fields (Jung and Park 2015), particularly, the strains *A. calcoaceticus* NRRL B551 and *A. calcoaceticus* 396.1 since they are able to transform OTA (De Bellis et al. 2015; Hwang and Draughon 1994). However, the *Acinetobacter* genus includes both non-pathogenic and pathogenic species; for instance, the *Acinetobacter baumannii*-*calcoaceticus* complex causes diverse human infections, the nosocomial ones affecting immunocompromised patients being especially relevant (Blossom and Srinivasan 2008; Zarrilli et al. 2023). Hence, it does not seem to be the most adequate bacterial species for biotechnological applications in the food industry. A comparable scenario is found for the *Acinetobacter* sp. *neg1* strain: although it is also capable of degrading OTA (De Bellis et al. 2015), shows the closest match to *A. gyllenbergii* (Fanelli et al. 2015), a haemolytic species isolated from human clinical specimens (Nemec et al. 2009) that has been described to be the origin of resistance to aminoglycosides of therapeutic importance in *Acinetobacter* strains (Yoon et al. 2016). Curiously, the recombinant variant of the carboxypeptidase PJ15_1540 from *Acinetobacter* sp. *neg1* exhibited a much lower OTA-degrading activity than that of the host strain (Liuzzi et al. 2017).

In this study, we analyzed the OTA-degrading ability of the bacterial species *A. tandoii* (Carr et al. 2003), a non-pathogenic species isolated from sludge plants, and identified a new α/β hydrolase in *A. tandoii* DSM 14970^ T^ (*At*ABH) that degrades OTA by hydrolysing its amide bond, producing OTα and L-β-phenylalanine. Modelling of the structure of *At*ABH with AlphaFold2 and subsequent in silico molecular docking approaches permitted us to propose an OTA-binding mode to *At*ABH that reveals a substrate-binding pocket with two hydrophobic sub-pockets around a catalytic triad. Further structural similarity searches show unexpected relationships of *At*ABH with esterases, which has been verified experimentally revealing that *At*ABH is a promiscuous α/β hydrolase with an efficient esterase activity and a second, ancillary amidohydrolase activity. Based on these findings, we propose a structure-based roadmap for finding OTA-degrading enzymes.

## Materials and methods

### Bacterial strains and OTA-transformation assays

*Acinetobacter tandoii* DSM 14970^ T^ type strain used in this study was purchased from the Leibniz Institute DSMZ-German Collection of Microorganisms and Cell Cultures (DSMZ) and was routinely grown in TSB broth at 30 °C as recommended by DSMZ. *Escherichia coli* DH5α was used for DNA manipulations. *E. coli* BL21 (DE3) cells were used for protein expression in the pURI3-Cter vector (Curiel et al. 2011). *E. coli* strains were cultured in Luria–Bertani (LB) medium at 37 °C and shaking. When required, ampicillin was added to the medium at 100 µg/mL.

*A. tandoii* DSM 14970^ T^ cultures and cell-free protein extract were used to assay their ability to transform OTA. For the bacterial culture assays, *A. tandoii* was grown in minimal salt medium (MM) (Shabtai and Gutnick 1985), ethanol-minimal salt medium (pH 7.5; 2.5% ethanol; MME) (Hwang and Draughon 1994; Shabtai and Gutnick 1985), and minimal salt medium with Bacto Peptone (0.5% w/v) (MMP) (De Bellis et al. 2015). Cultures were grown for 6 days in the absence or in the presence of OTA (5 μM). Then, the cells were sedimented, and the supernatant was filtered (0.45 μm pore size; Sarstedt, Germany) and analyzed by HPLC for the presence of OTA as previously described (Sánchez-Arroyo et al. 2023).

The OTA transforming activity was also analyzed in cell-free protein extract. For extract preparation, *A. tandoii* DSM 14970^ T^ cells grown in TSB broth for 24 h were harvested by centrifugation (8000 × g for 15 min at 4 °C), washed with 50 mM MOPS buffer, pH 7.0 containing 20 mM NaCl, and resuspended in the buffer used for cell rupture. Bacterial cells were disintegrated by using a French press at 1100 psi pressure (Amicon French pressure cell, SLM Instruments). Cell disruption steps were carried out on ice to ensure the low-temperature conditions required for most enzymes. To remove cell debris, the cell suspension was centrifuged at 4 °C (12,000 × g for 20 min). To prepare the final protein extract, the clean supernatant was filtered with sterile filters of 0.45 μm pore size (Sarstedt, Germany). For OTA degradation assays, this protein extract was incubated in the presence of OTA (5 μM final concentration) overnight at 37 °C. Reactions were stopped by heating at 95 °C for 5 min. Subsequently, samples were centrifuged at 14,000 × *g* for 5 min. The supernatants were filtered through 0.45 μm syringe filters (Millipore, USA) and diluted 1:1 in water/acetonitrile/glacial acetic acid (89:10:1, *v*/v)], and analyzed by HPLC as previously described (Sánchez-Arroyo et al. 2023). Controls without proteins were analyzed to evaluate the spontaneous degradation of OTA. Identification of the degradation product as OTα was carried out by comparing its retention time and spectral data with those of OTα from a commercial supplier (LGC Standard, UK).

### Production and purification of an α/β hydrolase from *A.tandoii*

In the *A. tandoii* DSM 14970^ T^ genome, the gene encoding a putative α/β hydrolase in the locus Q340_RS0119210 (*At*ABH) was amplified by PCR using primers 1965 (5′-*AACTTTAAGAAGGAGATATACATatg*aaaccactgatccactttgcgca) and 1966 (5′-*GCTATTAATGATGATGATGATGATG*ttgcctgaatcaactgcttaatcaa) (italics: nucleotides pairing the expression vector; lowercase letters: nucleotides pairing the gene sequence). The 795-bp purified PCR product was inserted into the pURI3-Cter vector as described previously (Curiel et al. 2011). The resultant recombinant protein variants have a six-histidine affinity tag in their C-termini. *E. coli* DH5α cells were used for preparing the plasmids that were then transformed into *E. coli* BL21(DE3) cells. Protein production and purification of the His-tagged AtABH was carried out essentially as described before (Sánchez-Arroyo et al. 2023). Briefly, *E. coli* cells carrying the recombinant plasmid pURI3-Cter-ABH were grown at 22 °C in LB medium and induced with 0.25 mM isopropyl-β-D-thiogalactopyranoside (IPTG) (Ackerley et al. 2004). The cell lysate obtained by disruption with a French press was centrifuged at 47,000 × g for 40 min at 4 °C to remove the insoluble fraction. *At*ABH was purified by IMAC using TALON superflow resin (Clontech). The eluted His-tagged *At*ABH was dialyzed overnight at 4 °C against 50 mM sodium phosphate buffer, pH 7.0, containing 300 mM NaCl. The purity of the enzyme was checked by SDS-PAGE.

### Enzymatic degradation of OTA, OTB, and 4MF

Degradation of the mycotoxins OTA and ochratoxin B (OTB) was followed by HPLC. Stock solutions of OTA and OTB at 1 mg/mL concentration were prepared by dissolving them in methanol and stored at − 20 °C. The enzymatic hydrolysis reactions were conducted essentially as described previously, with minor modifications (Sánchez-Arroyo et al. 2023).

Conversely, we also used the OTA analog *N*-(4-methoxyphenylazoformyl)-phenylalanine (4MF) (Bachem, Switzerland) as substrate, to compare the degradation activity of purified *At*ABH with the commercial CPA (Sigma-Aldrich, Germany). Hydrolysis of 4MF was assayed as described (Sánchez-Arroyo et al. 2023).

### *At*ABH activity on synthetic carboxypeptidase substrates

The specificity profile of *At*ABH was analyzed by using a set of synthetic carboxypeptidase substrates with different amino acid residues at their C-terminal position. We have classified the substrates tested into three different families according to their chemical composition (Fig. S1 in the Supplementary information). Family I is composed of carbobenzyloxy-β-alanyl-L-alanine (ZAA); this compound is the only substrate containing a β-amino acid. Family II is composed of six potential carbobenzyloxy-derived substrates: carbobenzyloxy-L-alanine (ZA), carbobenzyloxy-L-alanyl-L-leucine (ZAL), carbobenzyloxy-L-alanyl-L-phenylalanine (ZAF), carbobenzyloxy-L-isoleucyl-L-phenylalanine (ZIF), carbobenzyloxy-L-phenylalanine (ZF), and carbobenzyloxy-L-phenylalanyl-L-isoleucine (ZFI). As can be observed, this family contains potential substrates with one (ZA, and ZF) or two (ZAL, ZAF, ZIF, ZFI) amino acids, together with the N-terminal carbobenzyloxy moiety. Finally, family III is made up of the hippuryl-derived compounds: hippuryl-phenylalanine (N-benzoyl-glycyl-phenylalanine) (HF), and hippuryl-arginine (N-benzoyl-glycyl-arginine) (HR). All these substrates were purchased from Sigma-Aldrich (Germany) and were manipulated as described previously (Sánchez-Arroyo, et al. 2023).

### Esterase enzymatic assay

The esterase activity of *At*ABH was determined by a spectrophotometric method using *p*-nitrophenyl esters of different chain lengths (Sigma-Aldrich) as substrates: *p*-nitrophenyl acetate (C2), *p*-nitrophenyl butyrate (C4), *p*-nitrophenyl caprylate (C8), *p*-nitrophenyl laurate (C12), *p*-nitrophenyl myristate (C14), and *p*-nitrophenyl palmitate (C16), as described previously (Esteban-Torres et al. 2013). Stock solutions (25 µM) of the *p*-nitrophenyl esters were prepared in acetonitrile-isopropanol (1/4, v/v).

The enzymatic reactions were done in 50 mM sodium phosphate buffer (pH 7.0) containing the substrate at 0.5 mM final concentration, and 30 µg of *At*ABH (3.8 µM). After 10 min of incubation at 37 °C, the reaction was stopped by chilling on ice, and the amount of *p*-nitrophenol released was determined as described before (Esteban-Torres et al. 2013). Enzyme assays were performed in triplicate.

### Molecular docking studies

Molecular docking was performed with the docking program AutoDock Vina (Forli et al. 2016) using UCSF Chimera 1.17 (Pettersen et al. 2004) as interface. We used the predicted structure of *At*ABH with AlphaFold2 (Jumper et al. 2021) as the receptor. Before its use in docking assays, the PDB file from AlphaFold2 was processed with Phenix (Liebschner et al. 2019), first with *phenix.process_predicted_model,* and then with *phenix.geometry_minimization*. The final, minimized model of *At*ABH was checked with Molprobity (Williams et al. 2018) and it showed excellent stereochemistry. In this regard, as expected for a canonical nucleophile serine residue of α/β hydrolases such as *At*ABH, its predicted catalytic serine residue (Ser75) is an outlier in the Ramachandran plot (there are 3 outliers out of 262 amino acids). The other two outlier residues are Pro35 and Phe225, which are far from the active site of *At*ABH and therefore are not relevant in the docking assays. On the other hand, the atomic coordinates of OTA (PubChem CID: 442,530) and OTB (PubChem CID: 20,966) were taken from the PubChem database (https://pubchem.ncbi.nlm.nih.gov/). The atomic coordinates for the OTA homolog 4MF were prepared with Chimera (Pettersen et al. 2004) by modifying those of OTA. The preparation of the structure of the receptor for docking assays involved the addition of polar hydrogens and the merging of the charges, and the preparation of the ligand involved the addition of hydrogens and Gasteiger charges. Additional parameters were: search exhaustiveness of 8, a maximum energy difference of 3 kcal/mol, and the number of binding modes was 10. The molecular docking was done using a grid box search space with dimensions *x* = 15 Å, *y* = 15 Å, and *z* = 15 Å centered at the coordinates of the oxygen atom of the Ser75 side chain. Evaluation of the best-docked poses was carried out considering that the scissile amide bond must be in close proximity and in the correct orientation to the nucleophile Ser75 side chain. UCSF Chimera (Pettersen et al. 2004), COOT (Emsley et al. 2010), and PyMOL (Schrödinger and DeLano 2020) were used for the visualization of the structures, analysis of the interactions, and figure preparation.

Prediction of the structure of the carboxypeptidase PJ15_1540 from *Acinetobacter* sp. *neg1* was also done with AlphaFold2 (Jumper et al. 2021). The resultant PDB file was processed with Phenix (Liebschner et al. 2019) as described above for *At*ABH. Structural similarity searches were done with the DALI Protein Structure Comparison Server (Holm 2022) (http://ekhidna2.biocenter.helsinki.fi/dali/). Pairwise structural superpositions were done with FATCAT (Li et al. 2020). Analyses of protein sequences for the presence of signal peptides were done with SignalP—6.0 (Almagro Armenteros et al. 2019) (https://services.healthtech.dtu.dk/services/SignalP-6.0/).

## Results

### Ochratoxin transformation by* A. tandoii *DSM 14970^T^

We observe that *A. tandoii* DSM 14970^ T^ was unable to grow in minimal-salts media devoid of carbon source, even in the presence of OTA. However, evident growth was detected when it was cultured in minimal media containing ethanol or peptone, regardless of the presence of OTA. Supernatants from these OTA-containing cultures were analyzed by HPLC for the presence of this mycotoxin. Although a slight reduction in OTA concentration with respect to controls was observed, the majority of OTA remained untransformed in the culture supernatant (data not shown), so it cannot be ruled out that the reduction observed could be due to degradation or a phenomenon of OTA adsorption to the bacterial cell walls.

**Fig. 1:**
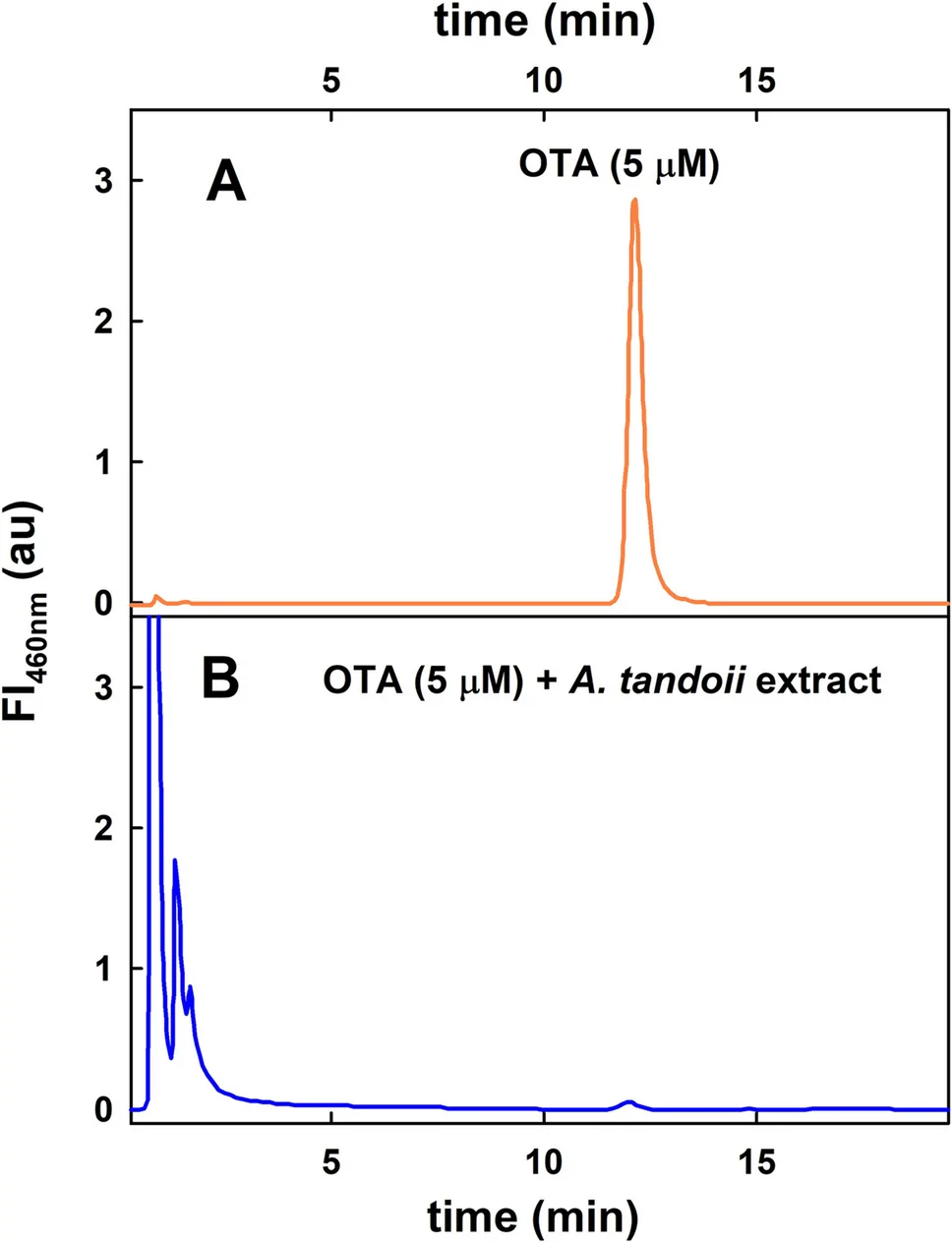
Amidase activity of *A. tandoii* DSM 14970^ T^ cell-free protein extract on OTA. HPLC chromatograms of *A. tandoii* extract incubated at 37 ºC for 16 h in the presence of OTA (5 μM). A control reaction without extract is indicated (A). The fluorescence wavelengths were 340 nm for excitation and 436 nm for emission

Since the results obtained with bacterial cultures from *A. tandoii* DSM 14970^ T^ were not conclusive, OTA degradation was assayed in a cell-free protein extract. As shown in Fig. 1, when protein extract from this strain was assayed, the OTA present in the reaction was hydrolysed, and the presence of OTα was detected in the *A. tandoii* protein extract reaction (Fig. 1). This result confirmed that *A. tandoii* DSM 14970^ T^ hydrolyses the amide bond of OTA, producing the innocuous products OTα and L-β-phenylalanine.

### OTA-hydrolyzing activity of an α/β hydrolase from *A.tandoii*

With the aim to identify putative OTA-degrading enzymes from *A. tandoii* DSM 14970^ T^, we performed a protein search in the *A. tandoii* DSM 14970^ T^ genome. In particular, we focused our attention on genes coding for hydrolases or lactamases since they have been predicted as good OTA-degrading candidates (Xu et al. 2021). From the proteins annotated as hydrolases in the *A. tandoii* DSM 14970^ T^ genome, the “α/β hydrolase” encoded in the locus Q340_RS0119210 (WP_026441294), herein referred as *At*ABH, was chosen to check its OTA-transforming activity because it is widely distributed in *Acinetobacter* species, including the *Acinetobacter* sp. *neg1* strain (locus PJ15_RS15480, WP_047432341) (Fig. S2 in the Supplementary information).

Hence, the gene encoding *At*ABH was cloned into the pURI3-Cter vector as described previously (Curiel et al. 2011). The C-terminally His-tagged *At*ABH protein thus produced was purified by IMAC chromatography. Fig. S3 in the Supplementary information shows that the band of purified *At*ABH in SDS-PAGE corresponded to the molecular size expected for the enzyme (≈30 kDa). This final production yield was 6.9 mg of *At*ABH per liter of culture.

**Fig. 2:**
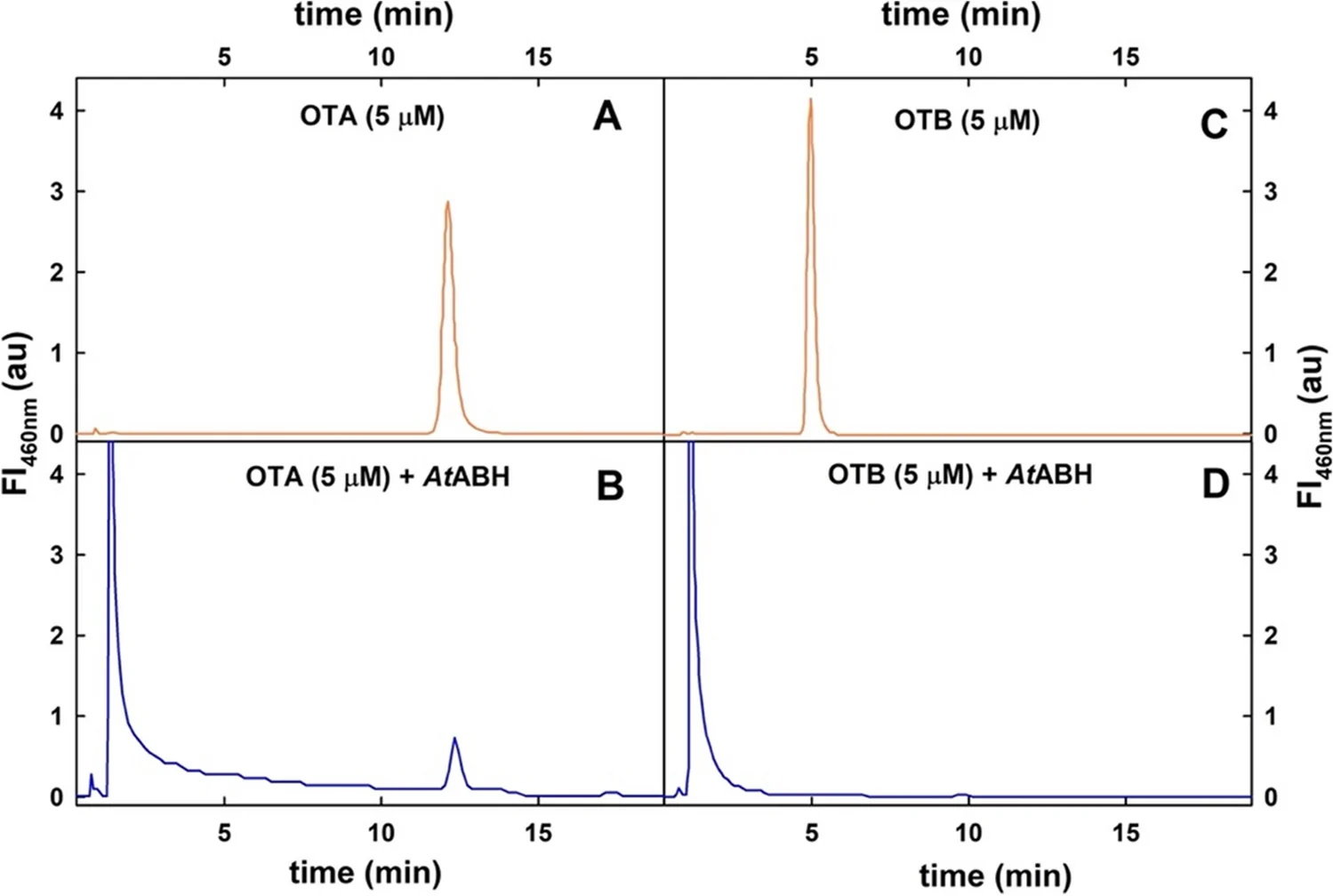
Amidase activity of Amidase activity of ABH from *A. tandoii* DSM 14970^ T^ on OTA (A) and OTB (B). HPLC chromatograms of *At*ABH incubated at 37 °C for 16 h in the presence of OTA (B) and OTB (D) at 5 μM concentration. Control reactions without *At*ABH are indicated (A and C). The fluorescence wavelengths were 340 nm for excitation and 436 nm for emission

Pure *At*ABH was incubated overnight in the presence of OTA or OTB (a nonchlorinated form of OTA), and then the reaction mixtures were analyzed by DAD-FL-HPLC. As shown in Fig. 2, both mycotoxins were hydrolysed, yielding the expected products. In particular, the molecular species with a retention time of 1.4 min (Fig. 2B) was identified as OTα since both the retention time and its spectral properties are identical to those from the commercial standard. Therefore, *At*ABH behaves as an amidohydrolase since it hydrolysed OTA producing OTα and L-β-phenylalanine.

### Analysis of the specificity profile of *At*ABH

**Fig. 3:**
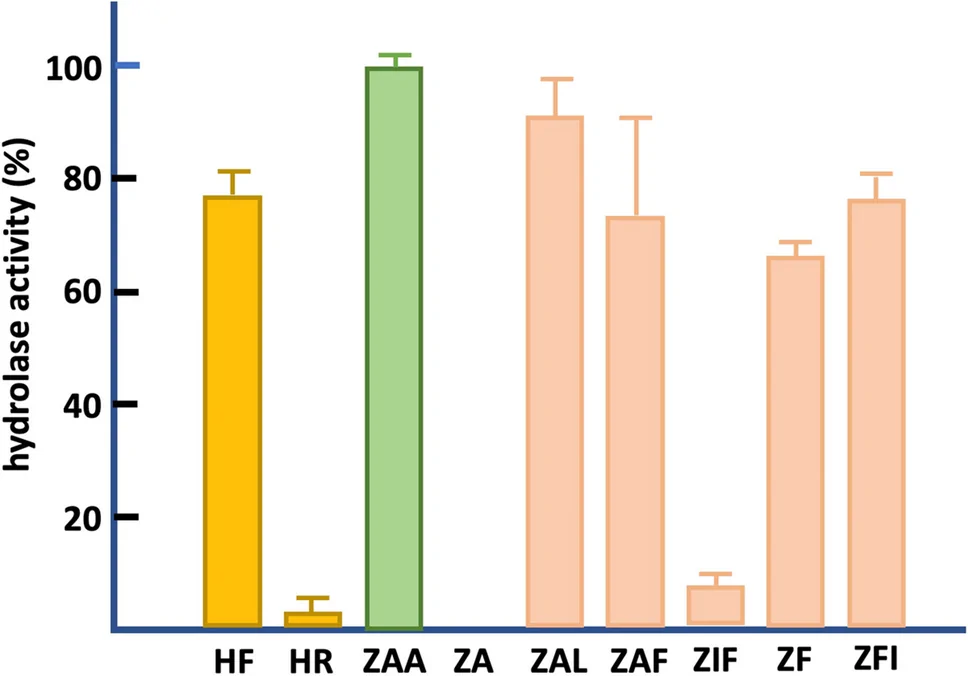
Substrate profile of *A. tandoii* ABH amidase activity on carboxypeptidase synthetic substrates harbouring different substitutions around the scissile amide bond. The substrates are HF, HR, ZAA, ZA, ZAL, ZAF, ZF, ZIF, and ZFI (see Fig. S1 in the Supplementary information for structural details). The error bars represent the standard deviation estimated from the three independent assays. The observed maximum activity was defined as 100%. Colour code is as in Fig. S1

To explore the hydrolase specificity profile of *At*ABH, we used three structural families of substrates (Fig. S1 in the Supplementary information), which permitted us to probe the effects on the hydrolase activity of different substituents at both the N- and the C-terminal sides around the scissile amide bond. In this regard, since OTA, OTB, as well as the OTA analog *N*-(4-methoxyphenylazoformyl)-phenylalanine 4MF (not shown) are hydrolysed by *At*ABH and all of them contain a phenylalanyl moiety at their C-terminal end and an aromatic ring at the N-terminal end, it is expected that *At*ABH exhibited a preference for hydrophobic moieties at both the N- and C-terminal sides of the amide bond, a fact that is well supported by our modelling studies about the OTA-binding mode to *At*ABH (see below). The results obtained with the family composed of the substrates HF and HR (Fig. 3) agree well with this hypothesis since the presence in HR of a large, cationic moiety at the C-terminal side of the amide bond makes this compound a poorly hydrolysed substrate (3%) when compared to HF that presents a Phe residue (74%). Nonetheless, the presence of aromatic rings at both ends of the structure of a (putative) substrate is not a necessary condition for efficient hydrolysis as can be deduced from ZAA (100%) that contains only one aromatic ring at the N-terminal end, a C-terminal methyl group and a β-alanyl bridge in the central part of the molecule (Fig. S1 in the Supplementary information). Yet, the combined absence of a central bridge and a C-terminal Phe, as in ZA, was crucial for hydrolysis since this compound was not hydrolysed (Fig. 3). Since all the rest of the compounds from the ZA family were efficiently hydrolysed (excluding ZIF, which has a rigid Ile residue within the central bridge), it can be deduced that, for members of this family, the presence of both a central bridge and a C-terminal aromatic/aliphatic residue are sufficient conditions to define a good substrate for *At*ABH. As indicated below, these results are in agreement with the in silico model of the structure of *At*ABH that predicts a substrate-binding pocket composed of two hydrophobic sub-pockets around its canonical catalytic triad.

### In silico structural model of *At*ABH

**Fig. 4:**
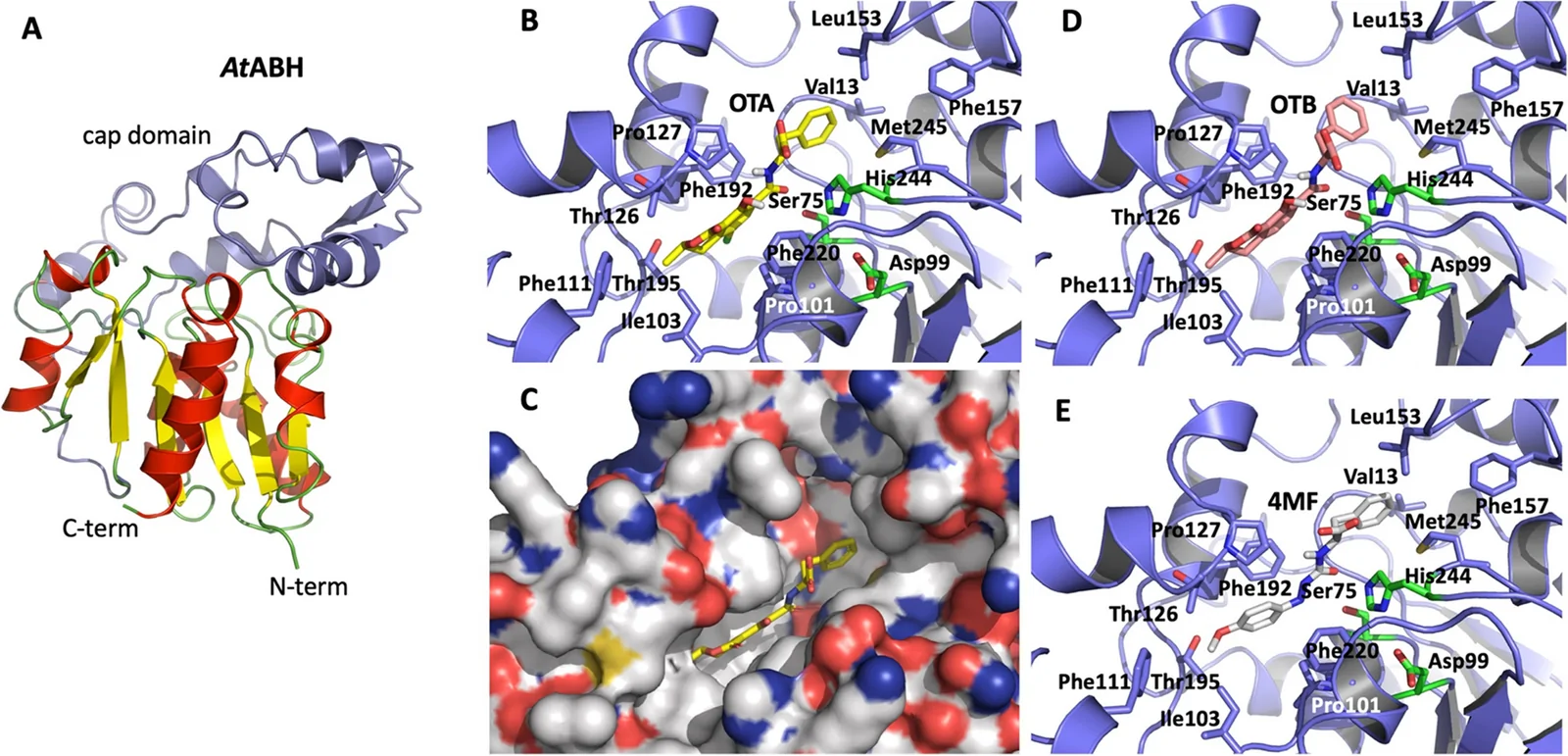
Predicted model of the structure of *At*ABH with AlphaFold2 and in silico molecular docking of ochratoxin A OTA, OTB, and 4MF (see Fig. S1 in the Supplementary information for structural details). The structure of *At*ABH (A) shows an αβ hydrolase fold formed by a parallel, six-stranded β-sheet (β-strands in *yellow*) surrounded by α-helices (in *red*) with a large cap domain (*blue ribbon*). The cap domain is comprised of the segment between residues Asp99 and Pro209 and is shown as *blue ribbon*. The OTA-binding mode (B, C) reveals that its amide bond is situated in close proximity to the nucleophile Ser75 that together with Asp99 and His244 made up the catalytic triad of *At*ABH (*green sticks*). The substrate-binding pocket is formed by a crevice (C) which has two hydrophobic sub-pockets around the nucleophile Ser75, where the two ends of the substrates bind. OTB (D) and 4MF (E) binding modes are almost identical to the one of OTA (B). Amino acid side chains that form the substrate-binding pocket are shown as *blue sticks* model. OTA, OTB, and 4MF are shown as *yellow*, *orange*, and *grey sticks*, respectively

In agreement with the genomic annotation of the locus Q340_RS0119210 from *A. tandoii* DSM 14970^ T^, the predicted structure of *At*ABH obtained with AlphaFold2 (Jumper et al. 2021) indicates that this enzyme belongs to the α/β hydrolase superfamily of enzymes (Heikinheimo et al. 1999; Nardini and Dijkstra 1999; Ollis et al. 1992). The predicted structure of *At*ABH indicates that when compared to the canonical α/β hydrolase fold, whose core structure is based on an eight-stranded mostly parallel β-sheet, *At*ABH lacks the first two, N-terminal canonical β-strands, and therefore its structural core is a parallel, six-stranded β-sheet (Fig. 4a). The catalytic machinery of *At*ABH supported by this structural framework, as it is typical in the α/β hydrolases (Rauwerdink and Kazlauskas 2015), is based on a catalytic triad made up of residues Ser75, Asp99, and His244 (Fig. 4b). The nucleophile Ser75 is situated in a Gly-Xxx-Ser-Xxx-Gly canonical sequence motif (73-Gly-His-Ser-Leu-Gly-77), which is predicted to adopt a sharp turn, known as nucleophile elbow, between strand β3 and helix α3. Conversely, His244 and Asp99 are located in the β9-α12 and the β4-α4 connecting loops, respectively. The large insertion between the β4 and β5 strands forming the cap domain of *At*ABH is made up of the segment between residues Asp99 and Pro209 that, as shown in Fig. 4a, configures the walls of the substrate binding pocket of the enzyme.

### OTA-binding mode

Following the same experimental procedure that we have recently used for the analysis of the OTA-binding mode to the salicylate 1,2-dioxygenase from *Pseudaminobacter salicylatoxidans* (Sánchez-Arroyo et al. 2023), we analyzed the binding mode of OTA, OTB, and 4MF to the predicted structure of *At*ABH. The grid box search space was centred around the residue Ser75, whose identification as the nucleophile was straightforward since, as indicated above, a catalytic triad defines the catalytic machinery of α/β hydrolases (Fig. 4b). The catalytic triad residues of *At*ABH (Ser75, Asp99, and His244) are situated at the bottom of a crevice (Fig. 4c) where the three substrates get access. The combined analysis of the docked poses of these three substrates permitted us to infer a consistent substrate-binding mode that places their corresponding scissile amide bonds in close proximity to the Ser75 side chain. As expected, the binding of OTA (Fig. 4b and c) and OTB (Fig. 4d) is almost identical: the (3R)-5-chloro-8-hydroxy-3-methyl-1-oxo-3,4-dihydroisochromene moiety of OTA (OTB lacks the 5-chloro substituent) is sandwiched between the side chains of the two pairs of residues Pro127, Phe192, and Pro101, Phe220, respectively, and it reaches a sub-pocket formed by the side chains of Ile103, Phe 111, Thr126, and Thr195. On the other hand, the aromatic ring of the Phe residue of OTA (or OTB) occupies another sub-pocket where it interacts with the Val13, Leu153, and Met245 side chains. Finally, the binding of 4MF places its amide bond in the same position and orientation observed for OTA or OTB, and its two aromatic rings occupy the two sub-pockets indicated above (Fig. 4e). The carboxylate moiety occupies the same location in all three cases and protrudes toward the solvent.

### Structural similarity searches

**Fig. 5:**
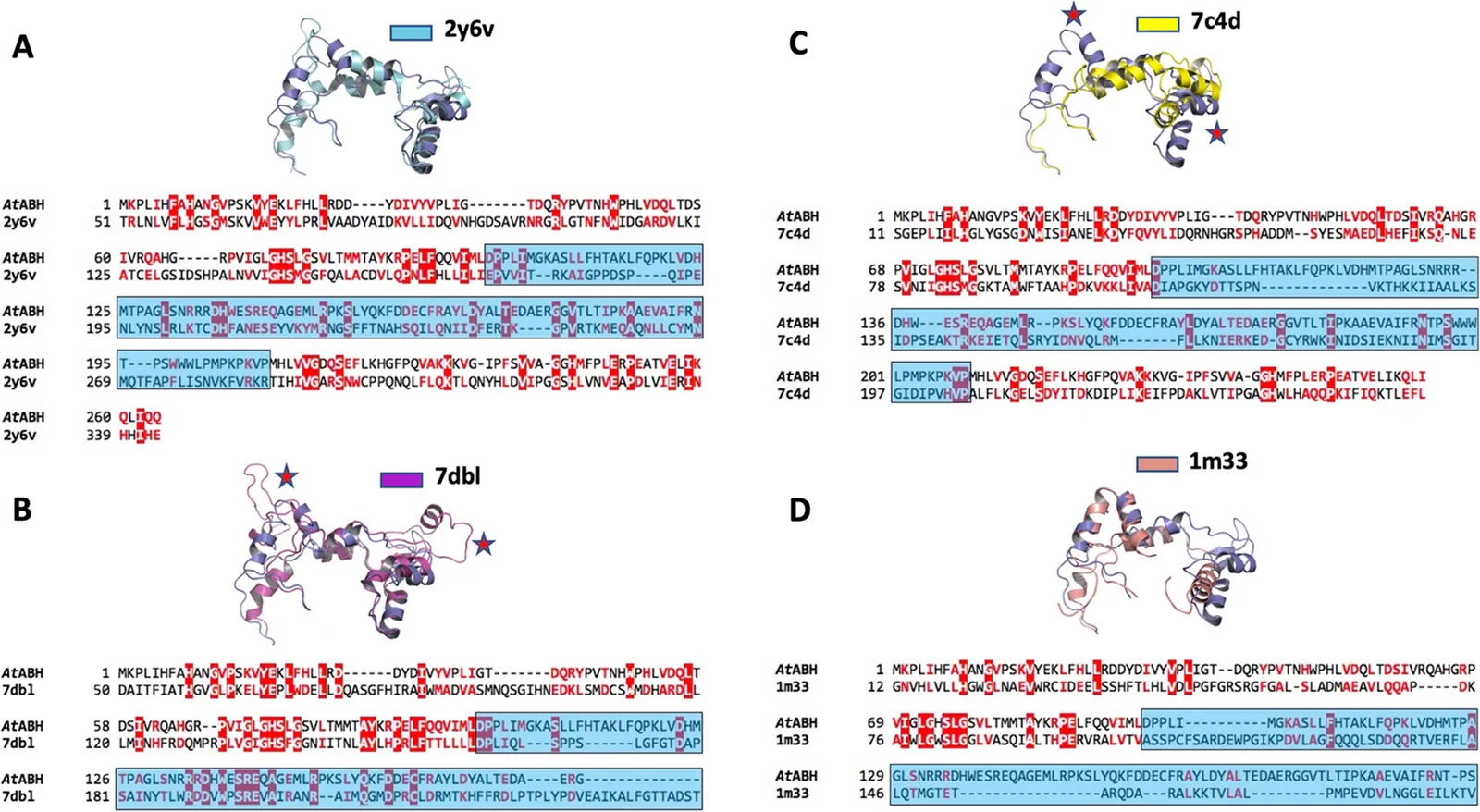
Analysis of the structural similarity between the cap domains of *At*ABH and its close homologs as determined with DALI. The cap domains of the αβ hydrolase Lpx1 from *Saccharomyces cerevisiae* (*cyan ribbon* model, A; PDB entry: 2y6v), the acyl-CoA hydrolase MpaH from *Penicillium brevicompactum* (*magenta ribbon* model, B; PDB entry: 7dbl), the marine esterase EprEst (*yellow ribbon* model, C; PDB entry: 7c4d), and the carboxylesterase BioH from *E. coli* (*orange ribbon* model, D; PDB entry: 1m33) are shown superimposed to the cap domain of *At*ABH (*blue ribbon* model). Structural differences are indicated with a red star. The corresponding structure-based sequence alignment is shown for each structural superposition. Conserved amino acids are shown with white, bold characters and red background and similar residues as red, bold characters

Once an OTA-binding mode compatible with the predicted structure of *At*ABH has been determined, we carried out structural similarity searches using the DALI server (Holm 2022) with the aim of finding other potential OTA-hydrolysing enzymes, structurally similar to *At*ABH. The results show that the hits with the highest Z-score values (see Table 1) not only presented the conserved α/β hydrolase fold but also very similar cap domains (Fig. 5).

**Table 1:** Structurally similar proteins to *At*ABH determined with DALI

Protein	Z-score	rmsd (Å)	lali^1^	%id^2^	Catalytic triad residues	Cap domain
*At*ABH	–	–	–	–	Ser75/His244/Asp99	Asp99-Pro209
2y6v	20.6	2.8	242	17	Ser145/His323/Glu169	Glu169-Arg284
7dbl	20.1	2.8	242	22	Ser139/His365/Asp163	Asp163-Arg315
7c4d	19.4	3.1	230	13	Ser85/His242/Asp109	Asp109-Pro205
1m33	19.1	2.7	210	14	Ser82/His235/Asp207	Ala106-Pro198

^1^lali, number of aligned residues; ^2^%id, sequence identity to *At*ABH

### Esterase activity in *At*ABH

**Fig. 6:**
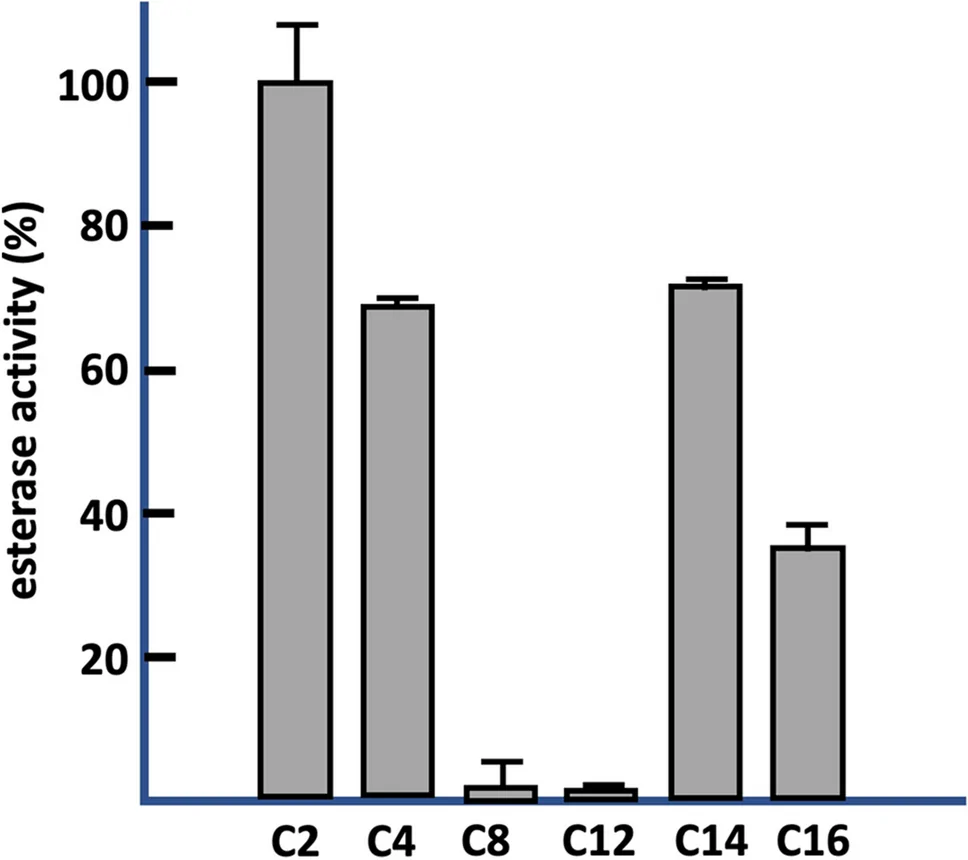
Substrate profile of *A. tandoii* ABH esterase activity on chromogenic substrates (*p*-nitrophenyl esters) with different acyl chain lengths (C2, acetate; C4, butyrate; C8, caprylate; C12, laurate; C14, myristate; C16, palmitate). The figure shows the relative specificities obtained toward different substrates. The error bars represent the standard deviation estimated from the three independent assays. The observed maximum activity was defined as 100%

Based on the structural similarity found between *At*ABH and the marine esterase EprEst (Zhu et al. 2021) (PDB entry: 7c4d; see Table 1) and also in the fact that many α/β hydrolases have multiple catalytic activities (Bauer et al. 2020), we consider the potential esterase activity of *At*ABH using different *p*-nitrophenyl (*p*NP) esters of various acyl chain lengths (C2 to C16) as substrates. Substrate specificity was assayed after a 10-min incubation at 37 °C. Our results indicate that *At*ABH hydrolysed water-soluble *p*-NP esters, with a marked preference for the shortest substrates C2 > C4 >  > C8 (Fig. 6), and remarkably, also *p*-NP myristate (C14) and *p*-NP palmitate (C16), long-chain, water-insoluble esters. Therefore, *At*ABH behaves as an esterase since it hydrolysed monomeric, water-soluble esters and as a lipase since hydrolysed aggregated, water-insoluble esters (Fojan et al. 2000).

## Discussion

A great variety of microorganisms have been reported to be able to transform OTA (Wang et al. 2022), among others some *Acinetobacter* species such as two strains from the *Acinetobacter calcoaceticus* species, namely, *A. calcoaceticus* NRL B551 and *A. calcoaceticus* 396.1 (De Bellis et al. 2015; Hwang and Draughon 1994) and also the strain *Acinetobacter* sp. *neg1* (De Bellis et al. 2015), that shows the closest match to *A. gyllenbergii* (Fanelli et al. 2015). Regarding *A. calcoaceticus* NRRL B-551, previous studies revealed that it did not grow in minimal-salts medium containing OTA, indicating that this strain was unable to use OTA as a sole carbon source (Hwang and Draughon 1994). However, when ethanol was added to the minimal-salts medium to provide a carbon source, it was able to grow and degrade OTA at 0.1 μg/mL/h at 30 °C (Hwang and Draughon 1994). Similarly, when peptone was used as the carbon source in the minimal-salts medium, *A. calcoaceticus* 396.1 and *Acinetobacter* sp. nov. strains also degrade OTA in aerobic conditions and at moderate temperatures (De Bellis et al. 2015). Since these OTA-transforming *Acinetobacter* species are related to clinical environments, in this work we focused our attention on the non-pathogenic strain *A. tandoii* DSM 14970^ T^ with the aim to evaluate its OTA-degrading capacity. Here we showed that cell-free protein extracts of *A. tandoii* DSM 14970^ T^ were competent in OTA degradation (Fig. 1), in contrast to bacterial cultures, which rendered inconclusive results.

In this sense, previous studies identified the carboxypeptidase PJ15_1540 from the *Acinetobacter* sp. *neg1* strain as an enzyme exhibiting hydrolytic activity against OTA. Although the recombinant variant of PJ15_1540 produced in *E. coli* BL21 (DE3) cells hydrolysed OTA, it was poorly efficient since it degraded a low percentage of the OTA present in the cell lysate (Liuzzi et al. 2017); in fact, the activity of this enzyme was much lower than that of the host strain. In this sense, PJ15_1540 is annotated as a D-alanyl-D-alanine carboxypeptidase and therefore potentially involved in the synthesis and remodelling of the peptidoglycan (Egan et al. 2020). In agreement with this, it has a signal peptide (residues Met1-Ala19), and the prediction of its structure with AlphaFold2 (Jumper et al. 2021) reveals an architecture based on an N-terminal D,D-carboxypeptidase domain, and an elongated C-terminal region (Fig. S4 in the Supplementary information), typical of penicillin-binding proteins (PBPs). In fact, the most structurally similar proteins are PBPs (Table S1 in the Supplementary information). A distinct characteristic of the proposed structure for PJ15_1540 is the presence of a large, amphipathic C-terminal helix (Fig. S4 in the Supplementary information), a structural motif involved in the association of PBPs with the membrane. We consider that this characteristic could make PJ15_1540 and other similar carboxypeptidases sub-optimal enzymes for biotechnological applications aimed at the degradation of OTA since it compromises their solubility. In fact, we think that the presence of this motif may explain the much lower OTA-degrading activity observed for recombinant PJ15_1540 versus the host strain (Liuzzi et al. 2017).

Likewise, similar carboxypeptidases from other sources such as *Bacillus amyloliquefaciens* ASAG1 (Chang et al. 2015), *Bacillus subtilis* CW14 (Hu et al. 2018; Xu et al. 2021), and *Lysobacter* sp. CW239 (Wei et al. 2020) also exhibited a low OTA-degrading activity. Conversely, the presence of OTA-degrading activity in a cp4 carboxypeptidase knock-out *Lysobacter* sp. CW239 strain (Qian et al. 2021) as well as in an amidohydrolase NA deficient *Stenotrophomonas* sp. CW117 strain (Chen et al. 2022) suggests that some strains could contain multiple enzymes for OTA degradation. In agreement with this idea, proteins possessing amidohydrolase (Chen et al. 2022; Dobritzsch et al. 2014; Luo et al. 2022; Zhang et al. 2019) or carboxypeptidase activity (Chang et al. 2015; Hu et al. 2018; Liuzzi et al. 2017; Wei et al. 2020; Xu et al. 2022) have been described elsewhere to be able to hydrolyse the amide bond present in the OTA molecule.

Although *A. tandoii* DSM 14970^ T^ possesses a close homolog of the carboxypeptidase PJ15_1540 from the *Acinetobacter* sp. *neg1* strain (Q340_RS0101740), that may contribute to the observed OTA hydrolysis exhibited by protein cell extracts of *A. tandoii* DSM 14970^ T^, we identified *At*ABH as an α/β hydrolase that hydrolyses OTA (Fig. 2).

The predicted structure of *At*ABH (Fig. 4) shows that it belongs to the α/β hydrolase superfamily of enzymes (Heikinheimo et al. 1999; Nardini and Dijkstra 1999; Ollis et al. 1992). The members of this superfamily exhibit an inner structural core based on an eight-stranded mostly parallel β-sheet surrounded on both sides by α-helices, which is decorated with additional structural elements. Typically, one such additional element is an insertion, highly variable both in size and in structure, between canonical β6 and β7 strands (β4 and β5 in *At*ABH) that configures the so-called cap domain (Alvarez et al. 2014; Bauer et al. 2020), and that together with other connecting loops between β-strands of the central β-sheet, is key in modulating the entrance of substrates into the binding site of the enzyme. In this regard, the observed hydrolytic activity of *At*ABH against OTA, OTB, and 4MF reveals that the conformation and dynamics of this cap domain are compatible with the binding of these substrates. Considering this point, we raised the hypothesis that searching for α/β hydrolases with cap domains similar to that of *At*ABH maybe a good starting point for a roadmap aimed at finding novel OTA-degrading enzymes.

The molecular docking studies with OTA, OTB, and 4MF provide a consistent binding mode common to the three substrates. This binding reveals the presence of two hydrophobic sub-pockets around the catalytic triad of *At*ABH. This result explains well the specificity profile of the enzyme. This latter specificity can be explained assuming that each end of the substrates (N- and C-terminal) binds to a hydrophobic sub-pocket, with their amide bond being located in close proximity to the nucleophile Ser75. Efficient binding would result from a balance between the binding of each end of the substrate to a hydrophobic sub-pocket and the structure of the internal bridge of the molecule between the N- and C-terminal ends. These requirements would not be satisfied by ZA which lacks an internal bridge and a C-terminal large hydrophobic or aromatic residue, HR which has a C-terminal large cationic residue, and ZIF that most probably does not satisfy steric requirements around the nucleophile due to the internal, rigid aliphatic side chain of an Ile residue (Fig. 3). As a whole, these structural results support the binding of the mycotoxins and carboxypeptidase substrates, and in particular, reveal that the conformation of the cap domain permits these substrates to get access into the binding pocket of *At*ABH that is configured by two hydrophobic sub-pockets. Interestingly, the structure of this cap domain is similar to the one from the esterase EprEst (PDB entry: 7c4d; see Table 1) as deduced from structural similarity searches (Fig. 5). This finding is somehow supported by the unexpected esterase activity of *At*ABH and in particular its dependence on the *p*-NP ester chain length that clearly paralleled the one observed for EprEst (Zhu et al. 2021).

These functional results revealing that *At*ABH has both amidohydrolase and esterase activities, indicate that *At*ABH is a promiscuous enzyme (Aharoni et al. 2005; Hult and Berglund 2007; Kourist et al. 2008), which is a relatively common characteristic of members from the αβ hydrolase superfamily most probably supported by the high versatility of the catalytic triad as catalytic machinery (Kourist et al. 2008; Rauwerdink and Kazlauskas 2015). In this sense, taking into account the time scales of the amidohydrolase and esterase reactions, and in the absence of kinetic analyses, it can be claimed that *At*ABH is operatively much more efficient as esterase than amidohydrolase, in agreement with the observation that promiscuous enzymes exhibit a main, native activity and a second much less efficient one (Aharoni et al. 2005). Thus, we can consider *At*ABH as an esterase with an ancillary amidohydrolase activity.

In summary, our biochemical and structural results indicate that *At*ABH is a promiscuous αβ hydrolase with a primary esterase activity and a secondary, less efficient amidohydrolase activity that endows *At*ABH with OTA-degrading capacity. The predicted substrate-binding pocket of *At*ABH reveals two hydrophobic sub-pockets around the nucleophile Ser75, which explains its specificity profile. Also, the prediction of the structure of the carboxypeptidase PJ15_1540 from *Acinetobacter* sp. *neg1* reveals a C-terminal amphipathic α-helix, most probably involved in the association with the membrane, which may explain previous results reporting low activity. Finally, we propose the following procedure for preparing OTA-degrading enzymes: (i) identification of promiscuous αβ hydrolases with OTA-degrading activity; (ii) prediction of their structures with AlphaFold2, followed by molecular docking studies to determine a plausible OTA-binding mode; (iii) identification and selection of proteins with similar cap domains and substrate-binding pockets; (iv) optimization of OTA-degrading activity through structure-guided preparation of protein variants.

## Supplementary Information

Below is the link to the electronic supplementary material.

Supplementary file1 (PDF 1955 KB)

## Data Availability

The authors declare that the data supporting the results of this study are available within the paper and its Supplementary information. Should any raw data be needed, they are available from the corresponding author upon reasonable request.

## References

[CR1] Abraham N, Chan ETS, Zhou T, Seah SYK (2022) Microbial detoxification of mycotoxins in food. Front Microbiol 13:1–18. 10.3389/fmicb.2022.957148 10.3389/fmicb.2022.957148 PMC9726736 36504774

[CR2] Ackerley DF, Gonzalez CF, Park CH, Blake R, Keyhan M, Matin A (2004) Chromate-reducing properties of soluble flavoproteins from *Pseudomonas putida* and *Escherichia coli*. Appl Environ Microbiol 70:873–882. 10.1128/AEM.70.2.873-882.2004 14766567 10.1128/AEM.70.2.873-882.2004 PMC348923

[CR3] Aharoni A, Gaidukov L, Khersonsky O, Gould SMQ, Roodveldt C, Tawfik DS (2005) The “evolvability” of promiscuous protein functions. Nat Genet 37:73–76. 10.1038/ng1482 15568024 10.1038/ng1482

[CR4] Almagro Armenteros JJ, Tsirigos KD, Sønderby CK, Petersen TN, Winther O, Brunak S, von Heijne G, Nielsen H (2019) SignalP 5.0 improves signal peptide predictions using deep neural networks. Nat Biotechnol 37:420–423. 10.1038/s41587-019-0036-z 30778233 10.1038/s41587-019-0036-z

[CR5] Alvarez Y, Esteban-Torres M, Cortés-Cabrera A, Gago F, Acebrón I, Benavente R, Mardo K, De Las Rivas B, Muñoz R, Mancheño JM (2014) Esterase LpEst1 from *Lactobacillus plantarum*: a novel and atypical member of the αβ hydrolase superfamily of enzymes. PLoS One 9:e92257. 10.1371/journal.pone.0092257 10.1371/journal.pone.0092257 PMC3963902 24663330

[CR6] Amézqueta S, González-Peñas E, Murillo-Arbizu M, López de Cerain A (2009) Ochratoxin A decontamination: a review. Food Control 20:326–333. 10.1016/j.foodcont.2008.05.017

[CR7] Bauer TL, Buchholz PCF, Pleiss J (2020) The modular structure of α/β-hydrolases. FEBS J 287:1035–1053. 10.1111/febs.15071 31545554 10.1111/febs.15071

[CR8] Blossom DB, Srinivasan A (2008) Drug-resistant *Acinetobacter baumannii-calcoaceticus* Complex. Inf Dis Clin Pr 16:1–3

[CR9] Carr EL, Kämpfer P, Patel BKC, Gürtler V, Seviour RJ (2003) Seven novel species of *Acinetobacter* isolated from activated sludge. Int J Syst Evol Microbiol 53:953–963. 10.1099/ijs.0.02486-0 12892111 10.1099/ijs.0.02486-0

[CR10] Chang X, Wu Z, Wu S, Dai Y, Sun C (2015) Degradation of ochratoxin A by *Bacillus amyloliquefaciens* ASAG1. Food Addit Contam - Part A Chem Anal Control Expo Risk Assess 32:564–571. 10.1080/19440049.2014.991948 25517039 10.1080/19440049.2014.991948

[CR11] Chen N, Fei Q, Luo H, Fang Z, Xiao Y, Du Z, Yu Z (2022) Isoenzyme N -Acyl-L-amino acid amidohydrolase NA increases ochratoxin A degradation efficacy of *Stenotrophomonas sp.* CW117 by enhancing amidohydrolase ADH3 stability. Microbiol Spectr 10:e02205-e2222 35924842 10.1128/spectrum.02205-22 PMC9430628

[CR12] Claeys L, Romano C, De Ruyck K, Wilson H, Fervers B, Korenjak M, Zavadil J, Gunter MJ, De Saeger S, De Boevre M, Huybrechts I (2020) Mycotoxin exposure and human cancer risk: a systematic review of epidemiological studies. Compr Rev Food Sci Food Saf 19:1449–1464. 10.1111/1541-4337.12567 33337079 10.1111/1541-4337.12567

[CR13] Curiel JA, De Las RB, Mancheño JM, Muñoz R (2011) The pURI family of expression vectors: a versatile set of ligation independent cloning plasmids for producing recombinant His-fusion proteins. Protein Expr Purif 76:44–53. 10.1016/j.pep.2010.10.013 21055470 10.1016/j.pep.2010.10.013

[CR14] De Bellis P, Tristezza M, Haidukowski M, Fanelli F, Sisto A, Mulè G, Grieco F (2015) Biodegradation of ochratoxin a by bacterial strains isolated from vineyard soils. Toxins (basel) 7:5079–5093. 10.3390/toxins7124864 26633497 10.3390/toxins7124864 PMC4690114

[CR15] Del Prete V, Rodriguez H, Carrascosa AV, De Las RB, Garcia-Moruno E, Muñoz R (2007) In vitro removal of ochratoxin A by wine lactic acid bacteria. J Food Prot 70:2155–2160. 10.4315/0362-028X-70.9.2155 17900096 10.4315/0362-028x-70.9.2155

[CR16] Schrödinger L, DeLano WL (2020) *PyMOL.* Available at: http://www.pymol.org/pymol

[CR17] Dobritzsch D, Wang H, Schneider G, Yu S (2014) Structural and functional characterization of ochratoxinase, a novel mycotoxin-degrading enzyme. Biochem J 462:441–452. 10.1042/BJ20140382 24947135 10.1042/BJ20140382

[CR18] Egan AJF, Errington J, Vollmer W (2020) Regulation of peptidoglycan synthesis and remodelling. Nat Rev Microbiol 18:446–460. 10.1038/s41579-020-0366-3 32424210 10.1038/s41579-020-0366-3

[CR19] Emsley P, Lohkamp B, Scott WG, Cowtan K (2010) Features and development of Coot. Acta Crystallogr Sect D Biol Crystallogr 66:486–501. 10.1107/S0907444910007493 20383002 10.1107/S0907444910007493 PMC2852313

[CR20] Esteban-Torres M, Reverón I, Mancheño JM, De las Rivas B, Muñoz R (2013) Characterization of a feruloyl esterase from *Lactobacillus plantarum*. Appl Environ Microbiol 79:5130-5136. 10.1128/AEM.01523-13 10.1128/AEM.01523-13 PMC3753946 23793626

[CR21] Fanelli F, Chiara M, Liuzzi VC, Haidukowski M, Tristezza M, Caterina M, D’Erchia AM, Pesole G, Horner DS, Mule G (2015) Draft genome sequence of *Acinetobacter sp. neg1* capable of degrading ochratoxin A. FEMS Microbiol Lett 362:1–4. 10.1093/femsle/fnv004 10.1093/femsle/fnv004 25790508

[CR22] Fojan P, Jonson PH, Petersen MTN, Petersen SB (2000) What distinguishes an esterase from a lipase: a novel structural approach. Biochimie 82:1033–1041. 10.1016/S0300-9084(00)01188-3 11099800 10.1016/s0300-9084(00)01188-3

[CR23] Forli S, Huey R, Pique ME, Sanner MF, Goodsell DS, Olson AJ (2016) Computational protein-ligand docking and virtual drug screening with the AutoDock suite. Nat Protoc 11:905–919. 10.1038/nprot.2016.051 27077332 10.1038/nprot.2016.051 PMC4868550

[CR24] Gan F, Zhou Y, Hou L, Qian G, Chen X, Huang K (2017) Ochratoxin A induces nephrotoxicity and immunotoxicity through different MAPK signaling pathways in PK15 cells and porcine primary splenocytes. Chemosphere 182:630–637. 10.1016/j.chemosphere.2017.05.030 28527416 10.1016/j.chemosphere.2017.05.030

[CR25] Heikinheimo P, Goldman A, Jeffries C, Ollis DL (1999) Of barn owls and bankers: a lush variety of α/β hydrolases. Structure 7:141–146. 10.1016/S0969-2126(99)80079-3 10.1016/s0969-2126(99)80079-3 10404588

[CR26] Holm L (2022) Dali server : structural unification of protein families. Nucleic Acids Res 50:W210–W215 35610055 10.1093/nar/gkac387 PMC9252788

[CR27] Hu HN, Jia X, Wang YP, Liang ZH (2018) Removal of ochratoxin A by a carboxypeptidase and peptides present in liquid cultures of *Bacillus subtilis* CW14. World Mycotoxin J 11:559–570. 10.3920/WMJ2017.2296

[CR28] Hult K, Berglund P (2007) Enzyme promiscuity: mechanism and applications. Trends Biotechnol 25:231–238. 10.1016/j.tibtech.2007.03.002 17379338 10.1016/j.tibtech.2007.03.002

[CR29] Hwang C-A, Draughon FA (1994) Degradation of ochratoxin A by *Acinetobacter calcoaceticus*. J Food Prot 57:410–414. 10.4315/0362-028x-57.5.410 31121738 10.4315/0362-028X-57.5.410

[CR30] Jumper J, Evans R, Pritzel A, Green T, Figurnov M, Ronneberger O, Tunyasuvunakool K, Bates R, Žídek A, Potapenko A, Bridgland A, Meyer C, Kohl SAA, Ballard AJ, Cowie A, Romera-Paredes B, Nikolov S, Jain R, Adler J, Back T, Petersen S, Reiman D, Clancy E, Zielinski M, Steinegger M, Pacholska M, Berghammer T, Bodenstein S, Silver D, Vinyals O, Senior AW, Kavukcuoglu K, Kohli P, Hassabis D (2021) Highly accurate protein structure prediction with AlphaFold. Nature 596:583–589. 10.1038/s41586-021-03819-2 34265844 10.1038/s41586-021-03819-2 PMC8371605

[CR31] Jung J, Park W (2015) Acinetobacter species as model microorganisms in environmental microbiology: current state and perspectives. Appl Microbiol Biotechnol 99:2533–2548. 10.1007/s00253-015-6439-y 25693672 10.1007/s00253-015-6439-y

[CR32] Kourist R, Bartsch S, Fransson L, Hult K, Bornscheuer UT (2008) Understanding promiscuous amidase activity of an esterase from *Bacillus subtilis*. ChemBioChem 9:67–69. 10.1002/cbic.200700521 18022973 10.1002/cbic.200700521

[CR33] Li Z, Jaroszewski L, Iyer M, Sedova M, Godzik A (2020) FATCAT 2.0: towards a better understanding of the structural diversity of proteins. Nucleic Acids Res 48:W60–W64. 10.1093/nar/gkaa443 32469061 10.1093/nar/gkaa443 PMC7319568

[CR34] Liebschner D, Afonine PV, Baker ML, Bunkoczi G, Chen VB, Croll TI, Hintze B, Hung L-W, Jain S, McCoy AJ, Moriarty NW, Oeffner RD, Poon BK, Prisant MG, Read RJ, Richardson JS, Richardson DC, Sammito MD, Sobolev OV, Stockwell DH, Terwilliger TC, Urzhumtsev AG, Videau LL, Williams CJ, Adams PD (2019) Macromolecular structure determination using X-rays, neutrons and electrons: recent developments in Phenix. Acta Crystallogr Sect d, Struct Biol D75:861–877. 10.1107/S2059798319011471 10.1107/S2059798319011471 PMC6778852 31588918

[CR35] Liuzzi VC, Fanelli F, Tristezza M, Haidukowski M, Picardi E, Manzari C, Lionetti C, Grieco F, Logrieco AF, Thon MR, Pesole G, Mulè G (2017) Transcriptional analysis of *Acinetobacter sp. neg1* capable of degrading ochratoxin A. Front Microbiol 7:1–9. 10.3389/fmicb.2016.02162 10.3389/fmicb.2016.02162 PMC5220012 28119679

[CR36] Luo H, Wang G, Chen N (2022) A superefficient Ochratoxin A hydrolase with promising potential for industrial applications. Appl Environ Microbiol 88:e01964-e2021. 10.1128/AEM.01964-21 34788069 10.1128/AEM.01964-21 PMC8788665

[CR37] Marin S, Ramos AJ, Cano-Sancho G, Sanchis V (2013) Mycotoxins: occurrence, toxicology, and exposure assessment. Food Chem Toxicol 60:218–237. 10.1016/j.fct.2013.07.047 23907020 10.1016/j.fct.2013.07.047

[CR38] McCoy AJ, Sammito MD, Read RJ (2022) Implications of AlphaFold2 for crystallographic phasing by molecular replacement. Acta Crystallogr Sect d, Struct Biol 78:1–13. 10.1107/S2059798321012122 34981757 10.1107/S2059798321012122 PMC8725160

[CR39] Nardini M, Dijkstra BW (1999) α/β hydrolase fold enzymes: the family keeps growing. Curr Opin Struct Biol 9:732–737. 10.1016/S0959-440X(99)00037-8 10607665 10.1016/s0959-440x(99)00037-8

[CR40] Ndiaye S, Zhang M, Fall M, Ayessou NM, Zhang Q, Li P (2022) Current review of mycotoxin biodegradation and bioadsorption: microorganisms, mechanisms, and main important applications. Toxins (Basel) 14:729. 10.3390/toxins14110729 10.3390/toxins14110729 PMC9694041 36355979

[CR41] Nemec A, Musílek M, Maixnerová M, De Baere T, van der Reijden TJK, Vannechoutte M, Dijkshoorn L (2009) *Acinetobacter beijerinckii* sp. nov. and *Acinetobacter gyllenbergii* sp. nov., haemolytic organisms isolated from humans. Int J Syst Evol Microbiol 59:118–124. 10.1099/ijs.0.001230-0 19126734 10.1099/ijs.0.001230-0

[CR42] Ollis DL, Cheah E, Cygler M, Dijkstra B, Frolow F, Franken SM, Harel M, Remington SJ, Silman I, Schrag J, Sussman JL, Verschueren KHG, Goldman A (1992) The α/β hydrolase fold. Protein Eng 5:197–211 1409539 10.1093/protein/5.3.197

[CR43] Pereira J, Simpkin AJ, Hartmann MD, Rigden DJ, Keegan RM, Lupas AN (2021) High-accuracy protein structure prediction in CASP14. Proteins 89:1687-1699. 10.1002/prot.26171 10.1002/prot.26171 34218458

[CR44] Pettersen EF, Goddard TD, Huang CC, Couch GS, Greenblatt DM, Meng EC, Ferrin TE (2004) UCSF Chimera - a visualization system for exploratory research and analysis. J Comput Chem 25:1605–1612. 10.1002/jcc.20084 15264254 10.1002/jcc.20084

[CR45] Pfohl-Leszkowicz A, Manderville RA (2007) Ochratoxin A: an overview on toxicity and carcinogenicity in animals and humans. Mol Nutr Food Res 51:61–99. 10.1002/mnfr.200600137 17195275 10.1002/mnfr.200600137

[CR46] Qian Y, Zhang X, Fei Q, Zhou Y (2021) Comments on the ochratoxin A degradation mechanism by *Lysobacter* sp CW239 — Wei Wei et al. (2020). Environ Pollut 281:117063. 10.1016/j.envpol.2021.117063 10.1016/j.envpol.2021.117063 33857714

[CR47] Rauwerdink A, Kazlauskas RJ (2015) How the same core catalytic machinery catalyzes 17 different reactions: the Serine-Histidine-Aspartate catalytic triad of alpha/beta-Hydrolase Fold Enzymes. ACS Catal 5:6153–6176. 10.1021/acscatal.5b01539 28580193 10.1021/acscatal.5b01539 PMC5455348

[CR48] Read RJ, Baker EN, Bond ChS, Garman EF, van Raaj MJ (2023) AlphaFold and the future of structural biology. Acta Crystallogr Sect d, Struct Biol 79:556–558. 10.1107/S20597983004928 37378959 10.1107/S2059798323004928

[CR49] Sánchez-Arroyo A, Plaza-Vinuesa L, Rivas B de las, Mancheño JM, Muñoz R (2023) The salicylate 1,2-dioxygenase from *Pseudaminobacter salicylatoxidans* DSM 6986T is a bifunctional enzyme that inactivates the mycotoxin ochratoxin A by a novel amidohydrolase activity. Int J Biol Macromol 237:124230. 10.1016/j.ijbiomac.2023.124230 10.1016/j.ijbiomac.2023.124230 36990411

[CR50] Shabtai Y, Gutnick DL (1985) Exocellular esterase and emulsan release from the cell surface of *Acinetobacter calcoaceticus*. J Bacteriol 161:1176–1181. 10.1128/jb.161.3.1176-1181.1985 3838301 10.1128/jb.161.3.1176-1181.1985 PMC215023

[CR51] Tunyasuvunakool K, Adler J, Wu Z, Green T, Zielinski M, Žídek A, Bridgland A, Cowie A, Meyer C, Laydon A, Velankar S, Kleywegt GJ, Bateman A, Evans R, Pritzel A, Figurnov M, Ronneberger O, Bates R, Kohl SAA, Potapenko A, Ballard AJ, Romera-Paredes B, Nikolov S, Jain R, Clancy E, Reiman D, Petersen S, Senior AW, Kavukcuoglu K, Birney E, Kohli P, Jumper J, Hassabis D (2021) Highly accurate protein structure prediction for the human proteome. Nature 596:590–596. 10.1038/S41586-021-03828-1 34293799 10.1038/s41586-021-03828-1 PMC8387240

[CR52] Wang L, Hua X, Shi J, Jing N, Ji T, Lv B, Liu L, Chen Y (2022) Ochratoxin A: occurrence and recent advances in detoxification. Toxicon 210:11–18. 10.1016/j.toxicon.2022.02.010 35181402 10.1016/j.toxicon.2022.02.010

[CR53] Wei W, Qian Y, Wu Y, Chen Y, Peng C, Luo M, Xu J, Zhou Y (2020) Detoxification of ochratoxin A by Lysobacter sp. CW239 and characteristics of a novel degrading gene carboxypeptidase cp4. Environ Pollut 258:113677. 10.1016/j.envpol.2019.113677 31843237 10.1016/j.envpol.2019.113677

[CR54] Williams CJ, Headd JJ, Moriarty NW, Prisant MG, Videau LL, Deis LN, Verma V, Keedy DA, Hintze BJ, Chen VB, Jain S, Lewis SM, Arendall WB, Snoeyink J, Adams PD, Lovell SC, Richardson JS, Richardson DC (2018) MolProbity: more and better reference data for improved all-atom structure validation. Protein Sci 27:293–315. 10.1002/pro.3330 29067766 10.1002/pro.3330 PMC5734394

[CR55] Xu H, Wang L, Sun J, Wang L, Guo H, Ye Y, Sun X (2022) Microbial detoxification of mycotoxins in food and feed. Crit Rev Food Sci Nutr 62:4951–4969. 10.1080/10408398.2021.1879730 33663294 10.1080/10408398.2021.1879730

[CR56] Xu X, Pang M, Liu J, Wang Y, Wu X, Huang KL, Liang Z (2021) Genome mining reveals the genes of carboxypeptidase for OTA-detoxification in *Bacillus subtilis* CW14. Int J Biol Macromol 186:800–810. 10.1016/j.ijbiomac.2021.07.085 34284053 10.1016/j.ijbiomac.2021.07.085

[CR57] Yoon EJ, Goussard S, Nemec A, Lambert T, Courvalin P, Grillot-Courvalin C (2016) Origin in *Acinetobacter gyllenbergii* and dissemination of aminoglycoside-modifying enzyme AAC(6’)-Ih. J Antimicrob Chemother 71:601–606. 10.1093/jac/dkv390 26645270 10.1093/jac/dkv390 PMC4743700

[CR58] Zarrilli R, Visca P, Bonnin RA, Dé E (2023) Editorial: drug resistance, global epidemiology and virulence of *Acinetobacter*. Front Microbiol 14:28–30. 10.3389/fmicb.2023.1151462 10.3389/fmicb.2023.1151462 PMC9989454 36896428

[CR59] Zhang H, Zhang Y, Yin T, Wang J, Zhang X (2019) Heterologous expression and characterization of a novel Ochratoxin A degrading enzyme, N-acyl-L-amino acid amidohydrolase, from *Alcaligenes faecalis*. Toxins (basel) 11:1–8. 10.3390/toxins11090518 10.3390/toxins11090518 PMC6784128 31489931

[CR60] Zhu C, Chen Y, Isupov MN, Littlechild JA, Sun L, Liu X, Wang Q, Gong H, Dong P, Zhang N, Wu Y (2021) Structural insights into a novel esterase from the East Pacific rise and its improved thermostability by a semirational design. J Agric Food Chem. 10.1021/acs.jafc.0c06338 33445864 10.1021/acs.jafc.0c06338

