# Supplementary material for: A new and promiscuous α/β hydrolase from Acinetobacter tandoii DSM 14970 T inactivates the mycotoxin ochratoxin A
Source: Appl Microbiol Biotechnol. 2024 Feb 23;108:230. doi: 10.1007/s00253-024-13073-x (PMC10891195; doi:10.1007/s00253-024-13073-x)
Supplement: Supplementary file 1 [file 253_2024_13073_MOESM1_ESM.pdf]

Supplementary Material

A new and promiscuous α/β hydrolase from *Acinetobacter tandoii* DSM 14970^T inactivates the mycotoxin ochratoxin A

Ana Sánchez-Arroyo[1], Laura Plaza-Vinuesa[1], María Claudia Abeijón-Mukdsi[1], Blanca de las Rivas[1], José Miguel Mancheño[2,*], and Rosario Muñoz[1,*]

[1] Institute of Food Science, Technology and Nutrition (ICTAN), CSIC, José Antonio Novais 6, 28040 Madrid, Spain
[2] Department of Crystallography and Structural Biology, Institute of Physical Chemistry Blas Cabrera, CSIC, Serrano 119, 28006 Madrid, Spain

*Corresponding authors:
Rosario Muñoz
r.munoz@csic.es

José Miguel Mancheño
jm.mancheno@csic.es

Figure S1

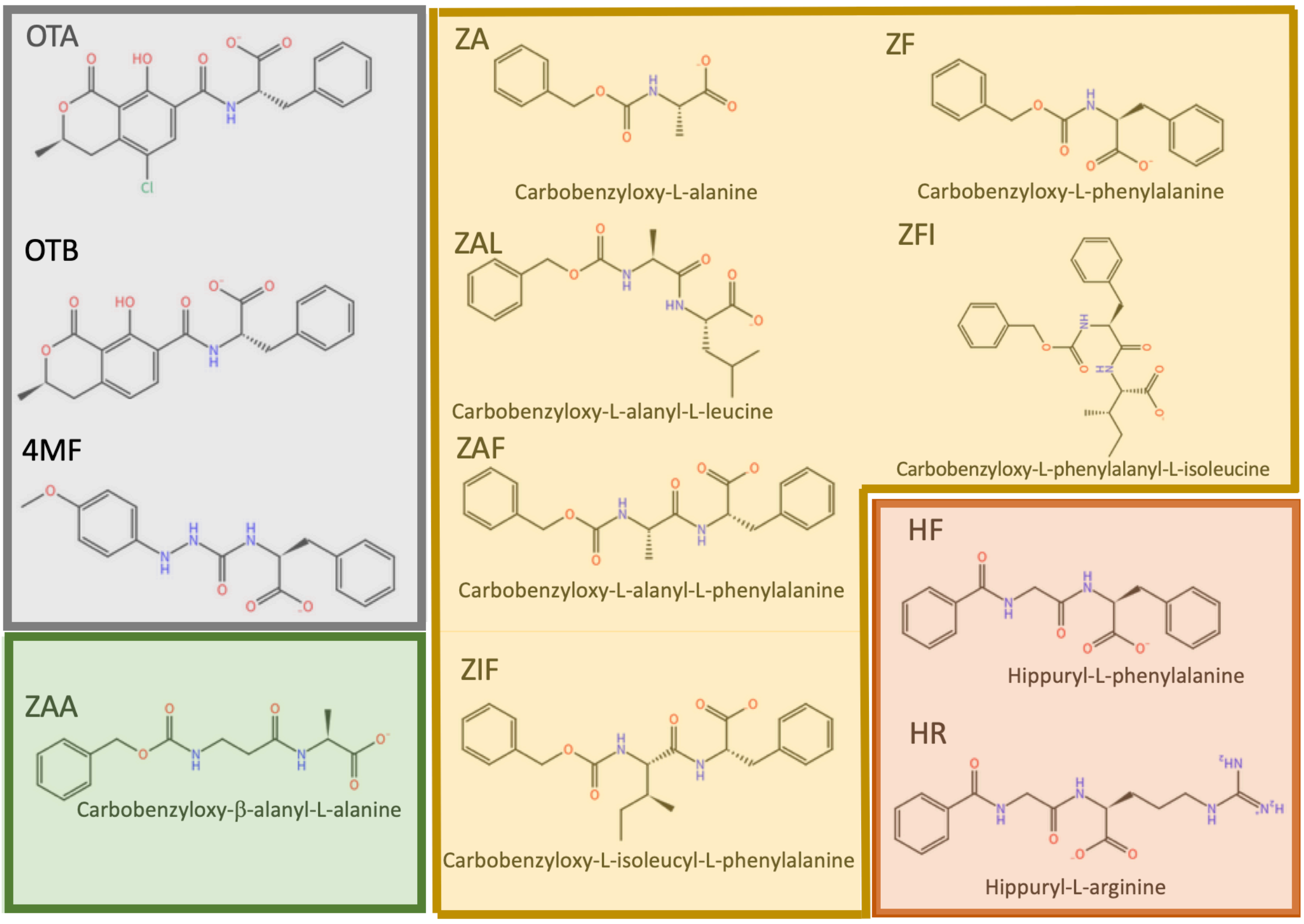

Fig. S1 2D structures of the synthetic carboxypeptidase substrates used in this work together with the structures of ochratoxins A and B, and its analog *N*-(4-methoxyphenylazoformyl)-phenylalanine (4MF).

Figure S2

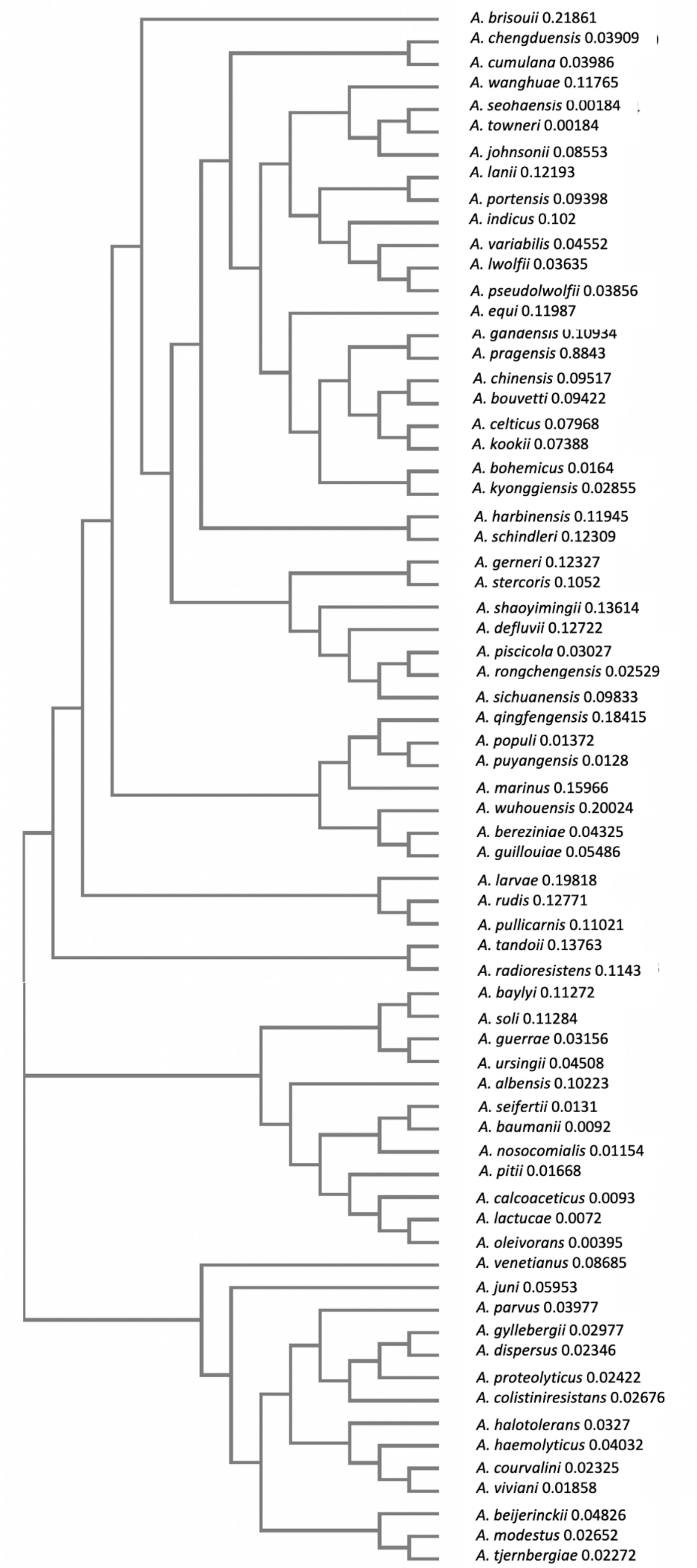

Fig. S2 Cladogram showing the distribution of the *At*ABH hydrolase within the *Acinetobacter* genus (69 different species). Distances between sequences were calculated with Clustal Omega Software.

Figure S3

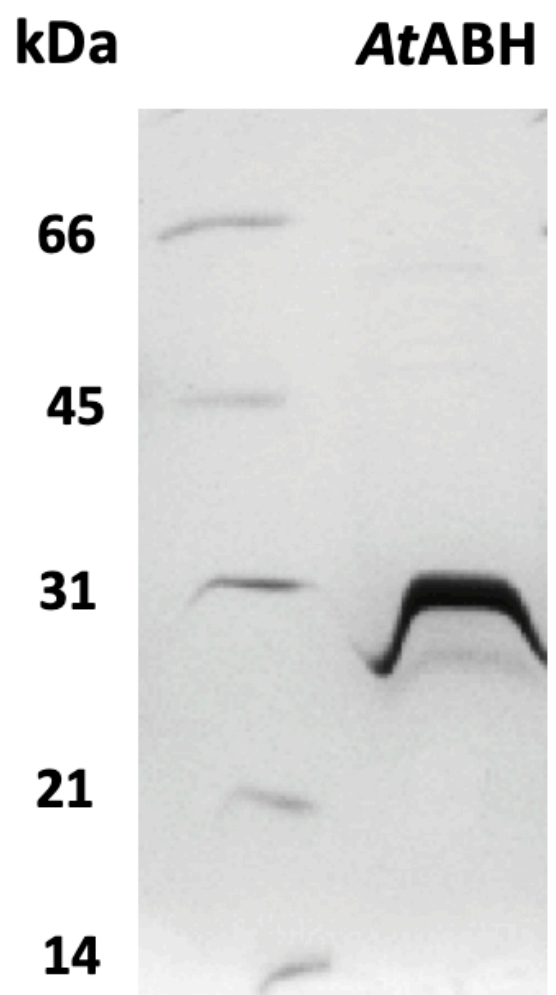

Fig. S3 SDS-PAGE analysis of the purification of *At*ABH recombinant protein. The 12.5% gel was stained with Coomassie blue. Molecular mass markers are located on the left (SDS-PAGE standards, Bio-Rad).

Figure S4

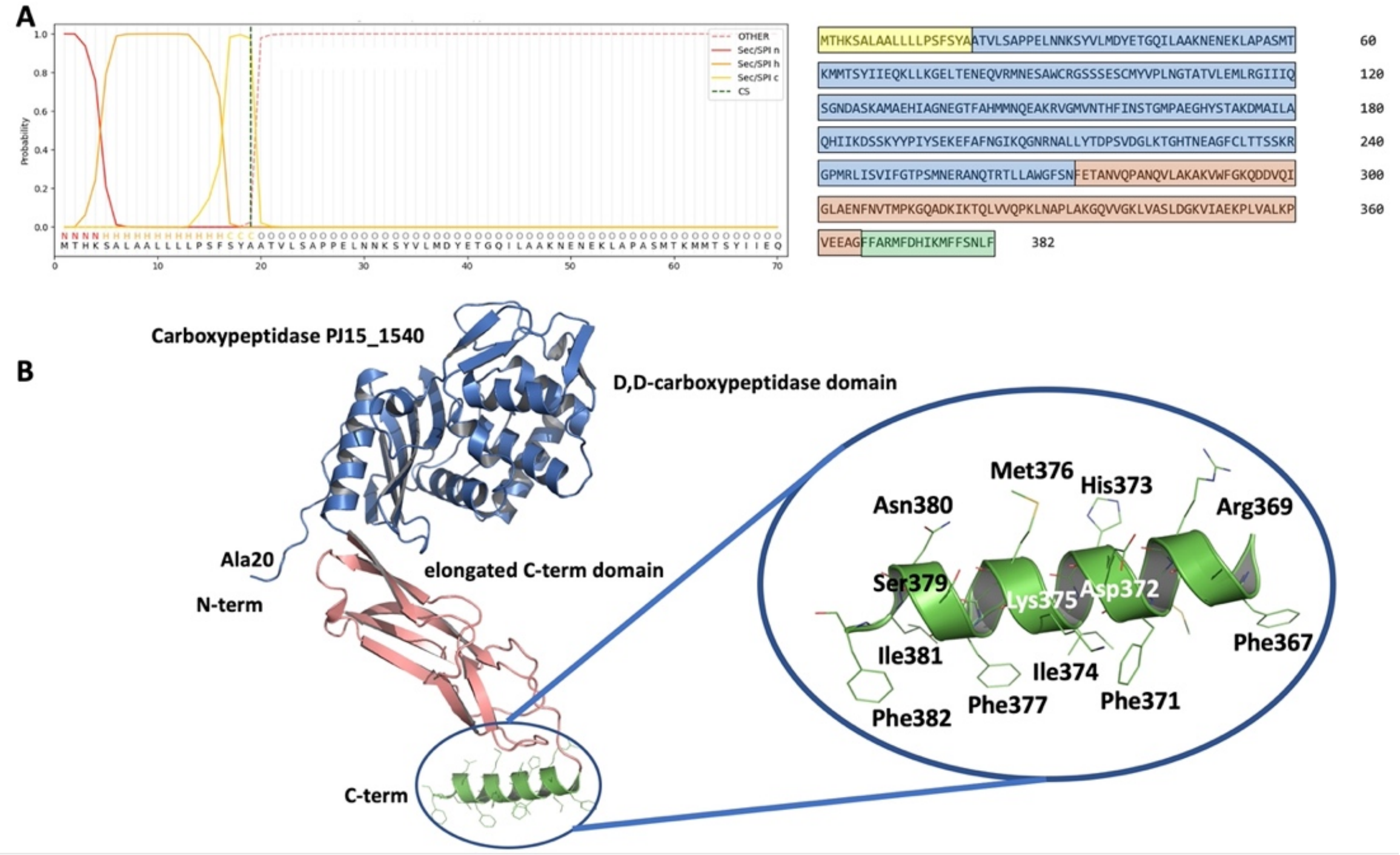

Fig. S4 Predicted structure of the carboxypeptidase PJ15_1540 from *Acinetobacter* sp. *neg1*. The structural model of PJ15_1540 obtained with AlphaFold2 reveals that the enzyme is a penicillin-binding protein with an N-terminal D,D-carboxypeptidase domain (*blue ribbon*), an elongated C-terminal domain (orange ribbon), and a C-terminal amphipathic α-helix (green ribbon). The close-up view of this helix remarks its markedly amphipathic character, which is consistent with the putative membrane-associated nature of the protein.

Table S1

Table S1. Structurally similar proteins to PJ15_1540 determined with DALI

Protein	Z-score	rmsd (Å)	lali[1]	%id[2]
**6osu**	34.0	1.3	251	41
**1xp4**	30.7	1.6	245	27
**1tvf**	29.6	1.8	247	30
**6muq**	26.2	2.3	239	24

[1]lali: number of aligned residues; [2]%id: sequence identity to PJ15_1540
